# *VEGF*, *VEGFR2* and *GSTM1* polymorphisms in outcome of multiple myeloma patients treated with thalidomide-based regimens

**DOI:** 10.1038/bcj.2017.58

**Published:** 2017-06-30

**Authors:** L Lopes-Aguiar, M T Delamain, A B C Brito, G J Lourenço, E F D Costa, G B Oliveira, J Vassallo, C A De Souza, C S P Lima

**Affiliations:** 1 Department of Internal Medicine, Faculty of Medical Sciences, Faculty of Medical Sciences, University of Campinas, Campinas, São Paulo, Brazil; 2 Haematology and Haemotherapy Centre, Faculty of Medical Sciences, University of Campinas, Campinas, São Paulo, Brazil; 3 Laboratory of Molecular and Investigative Pathology, Faculty of Medical Sciences, University of Campinas, Campinas, São Paulo, Brazil

Angiogenesis (AG) abnormalities are crucial in pathogenesis and prognosis of multiple myeloma (MM).^1^ Increased microvessel density (MVD) in bone marrow (BM) is an unfavorable prognostic factor in disease,^1^ supporting the use of inhibitors of vascular endothelial growth factor (VEGF) in patients’ treatment.^2^

VEGF and its VEGF type 2 receptor (VEGFR2),^1^ and hypoxia-inducible factor-1 alpha (HIF-1α)^3^ were described as key regulators of AG, and glutathione S-transferases mu1 (GSTM1) and theta1 (GSTT1) promotes AG by effecting the HIF-1α pathway.^4^ The wild-type alleles of *VEGF* c.-2595C>A (rs699947),^5^ c.-1154G>A (rs1570360),^5^ c.-634G>C (rs2010963),^6^ c.*237C>T (rs3025039),^7^ and *VEGFR2* c.-906T>C (rs2071559)^8^ and c.889G>A (rs2305948)^8^ single-nucleotide polymorphisms (SNPs) were associated with a higher production of VEGF or higher transcriptional activity and binding efficiency for VEGF than the respective variant alleles. On the other hand, *GSTM1* and *GSTT1* genes may be homozygous deleted in healthy individuals, having lack of respective active angiogenic proteins as a consequence.^9^

None of genotypes or haplotypes of *VEGF* SNPs (rs699947, rs833061, rs2010963 and rs3025039) have influenced in response to thalidomide of relapsed MM patients in a previous study.^10^ However, only the ACG haplotype of rs699947, rs833061 and rs2010963 loci, previously associated with higher production of VEGF,^5, 6^ altered negatively the time of thalidomide failure in those patients.^10^ *GSTM1* and *GSTT1* genes were previously described as unimportant in response and survival to vincristine, doxorubicin and dexamethasone (VAD) and high-dose melphalan in newly MM patients previous studied.^11^ However, worse disease-free survival and overall survival (OS) were related with the *GSTM1* present and *GSTT1* null genes in Hodgkin lymphoma patients.^12^

We investigated herein the roles of *VEGF* c.-2595C>A, c.-1154G>A, c.-634G>C, c.*237C>T, *VEGFR2* c.-906T>C, c.889G>A SNPs, and *GSTM1* and *GSTT1* genes, in outcome of MM patients treated with thalidomide-based regimens.

Newly diagnosed MM patients (*N*=102) were included in the study from June 2005 to June 2013, after local institutional review board guidelines approvals. Therapeutic regimens consisted in thalidomide combined with steroids and/or chemotherapy, followed or not by autologous stem cell transplantation (ASCT)^2^ (Supplementary Table S1). Fragments of BM available from diagnosis (*N*=21) served for immunohistochemistry analysis using anti-CD34 (QBEnd/10). Slides were scanned at × 20 magnification in Aperio Scanscope XT to assess MVD, in a blinded fashion.

Response was evaluated at the end of treatment using the International Myeloma Working Group guidelines, and classified as complete response (CR), very good partial response (VGPR), partial response (PR), stable disease (SD) or progressive disease (PD). Event-free survival (EFS) and OS encompassed time from diagnosis until relapse, progression, death due to tumor effects or last follow-up, and time from diagnosis until death by any cause or last follow-up, respectively.

Genotyping was performed in DNA of patients’ peripheral blood. *VEGF* and *VEGFR2* SNPs were analyzed by real-time polymerase chain reaction, using TaqMan SNP Genotyping Assays. Only the genotypes of *VEGF* c.*237C>T SNP, *GSTM1* and *GSTT1* genes were obtained by polymerase chain reaction plus enzymatic digestion and multiplex polymerase chain reaction, respectively.

The pairwise linkage disequilibrium was performed to ensure that markers were appropriate for inclusion in haplotype estimates. Two-tailed *t*-test was performed to investigate associations between genotypes and MVD. Logistic regression models assessed associations between genotypes and response. EFS and OS probabilities were estimated by Kaplan–Meier method and compared by log-rank test. The Cox hazards model was used to identify variables predicting EFS and OS. Variables with *P*⩽0.10 in univariate Cox analysis were included in multivariate Cox analysis. Significant results were validated using a bootstrap resampling study to investigate the stability of risk estimates (1000 replications). Differences were significant when *P*⩽0.05.

Linkage disequilibrium between *VEGF* and *VEGFR2* SNPs were seen in study, and common haplotypes (>1%) of the genes were included in further analyses.

MVD was higher only in patients with *VEGF* c.-1154GG genotype compared to others (8.64 × 10^−4^ vs 4.88 × 10^−4^ vessels/μm^2^, *P*=0.01) (Supplementary Figure S1).

Patients treated with thalidomide-based regimens followed by ASCT had more chances of achieving better response to therapy than others, and for this reason the values of logistic regression data were adjusted by ASCT status. The *VEGF* c.-2595CC or CA isolated or associated with *VEGFR2* c.-906TT or TC, and CGGC haplotype of *VEGF* c.-2595C>A, c.-1154G>A, c.-634G>C and c.*237C>T SNPs were also more common in patients with CR, VGPR or PR. Carriers of these genotypes or haplotype had 3.55, 9.91 and 3.86 more chances of obtaining better response to therapy, respectively (Table 1).

The median follow-up time of MM patients enrolled in study was 43 months. The estimated probabilities of 60-months EFS and OS were 24.5 and 48.1%, respectively. At the study end (February 2016), 50 patients were alive and 52 patients died.

In Kaplan–Meier estimates, the 60-months EFS and OS tended to be shorter in patients at ISS III (23.0 vs 25.2%, *P*=0.08; 41.3 vs 56.0%, *P*=0.08). At this time, both EFS and OS were shorter in patients who did not receive ASCT after chemotherapy (11.9 vs 42.4%, *P*<0.0001; 34.9 vs 65.1%, *P*<0.0001), with *VEGFR2* c.889GG (17.0 vs 43.5%, *P*=0.004; 42.2 vs 62.3%, *P*=0.03), *VEGF* c.-634GG plus *VEGFR2* c.889GG (22.8 vs 50.8%, *P*=0.01; 43.7 vs 85.7%, *P*=0.005), *VEGFR2* c.889GG plus *GSTM1* present (13.6 vs 31.6%, *P*=0.04; 30.7 vs 65.8%, *P*=0.01), respectively (Supplementary Figure S2). The *VEGF* c.-1154GG plus *VEGFR2* c.889GG (18.8 vs 42.1%, *P*=0.04) and *VEGFR2* c.-906TT plus c.889GG (13.3 vs 43.7%, *P*=0.001) predicted only worse EFS, and *GSTM1* present (39.0 vs 58.3%, *P*=0.09) and *VEGFR2* c.-906TT plus c.889GG (45.0 vs 56.4%, *P*=0.06) were marginally associated with shorter OS.

In univariate Cox analysis, the significance of differences between groups remained the same of the above analyses, and for this reason the values of multivariate Cox analysis were adjusted by ISS and ASCT status. Patients at stage III, patients who did not receive ASCT and those with the *VEGFR2* c.889GG, *VEGF* c.-1154GG plus *VEGFR2* c.889GG, *VEGF* c.-634GG plus *VEGFR2* c.889GG, *VEGFR2* c.-906TT plus c.889GG, and *VEGFR2* c.889GG plus *GSTM1* present genotypes had 1.66, 3.34, 2.62, 2.78, 2.64, 3.48 and 2.80 more chances of disease relapse or progression, respectively. Patients who did not receive ASCT, and those with the *VEGFR2* c.889GG, *GSTM1* present, *VEGF* c.-634GG plus *VEGFR2* c.889GG and *VEGFR2* c.889GG plus *GSTM1* present had 3.29, 2.21, 1.85, 4.88 and 4.23 more chances of evolving to death, respectively (Table 2).

We initially observed that carriers of *VEGF* c.-2595CC or CA genotype isolated or associated with *VEGFR2* c.-906TT or TC genotype, and the CGGC haplotype (rs699947, rs1570360, rs2010963 and rs3025039) of all analyzed *VEGF* SNPs, previously associated with higher VEGF effects,^5, 6, 7, 8^ presented better response to thalidomide-based regimens. In contrast, genotypes and haplotypes of *VEGF* SNPs (rs699947, rs833061, rs2010963 and rs3025039) did not influence the response to thalidomide in a unique study conducted in relapsed MM patients.^10^ Differences in response of tumors to thalidomide-based regimens may constitute a plausible explanation for the divergent results seen in both studies: only newly diagnosed MM patients were included in our study while that Andersen *et al.*^10^ analyzed only MM patients at relapse. On the other hand, *GSTM1* and *GSTT1* genes did not alter response to thalidomide-based regimens in our newly MM patients, and also in those previously treated with VAD and high-dose melphalan.^11^

Secondly, we found that carriers of *VEGF* c.-1154GG, *VEGF* c.-634GG, *VEGFR2* c.-906TT, *VEGFR2* c.889GG genotypes, and *GSTM1* present, alone or combined, previously associated with higher VEGF effects,^5, 6, 8^ had more chances of disease relapse/progression and/or of evolving to death. The genotypes of *VEGF* SNPs (rs699947, rs833061, rs2010963 and rs3025039) had no influence in survival of relapsed MM patients after thalidomide treatment in a previous study, but patients with the ACG haplotype of *VEGF* SNPs (rs699947, rs833061 and rs2010963 loci) presented a shorter time of thalidomide failure.^10^ On the other hand, no significant differences were observed in EFS and OS after VAD and high-dose melphalan in newly previously MM patients carrying or lacking the *GSTM1* gene.^11^ The disparate results obtained in both studies may be attributed to different types and doses of treatment used, as the first-line therapeutic regimens with conventional doses of thalidomide and ASCT in our study and intensive treatment with VAD and high-dose melphalan in the other study.^11^

In fact, VEGF plays an important role in tumor AG, acting as a potent inducer of vascular proliferation and permeability,^1^ and thus may advantage the action of therapy in MM tumor cells with consequently better response.^13^ However, VEGF also increases interleukin-6 secretion by endothelial and BM stromal cells, which stimulates MM cell growth, with consequent relapse of disease and death.^1^ We observed herein that BM of MM patients carrying the *VEGF* c.-1154GG genotype have increased MVD, and we have also recently shown that follicular lymphoma MVD was increased in patients with the CC genotype of *VEGF* c.-2595C>A SNP;^14^ these findings support associations between *VEGF* SNPs and MVD in lymphoproliferative disorders. In addition, *GSTM1* gene stimulates AG due to its effect on the HIF-1α metabolic pathway,^4^ and hyperexpression of HIF-1α was associated with MM progression.^15^

In summary, our data present, for the first time, a preliminary evidence that *VEGF* c.-2595C>A, c.-1154G>A, c.-634G>C, c.*237C>T, *VEGFR2* c.-906T>C, c.889G>A SNPs, and *GSTM1* gene, isolated or associated, alter outcome of newly diagnosed MM patients treated with conventional thalidomide-based regimens.

## Figures and Tables

**Table 1:** *VEGF*, *VEGFR2*, *GSTM1* and *GSTT1* polymorphisms in response rate of multiple myeloma patients

*Variable*	*Response rate (*N*=97)*^a^			
	*CR+VGPR+PR* N *(%)*	*SD+PD* N *(%)*	P-*value*	*OR* *(95% CI)*
*ISS*^a^				
I+II	40 (88.9)	5 (11.1)	0.25	Reference
III	41 (80.4)	10 (19.6)		1.97 (0.60–6.44)
				
*ASCT*				
Yes	40 (93.0)	3 (7.0)	0.05	Reference
No	42 (77.8)	12 (22.2)		3.74 (0.98–14.36)
				
*VEGF c.-2595C>A*				
CC	38 (84.4)	7 (15.6)	0.69	1.25 (0.39–3.93)
CA+AA	44 (84.6)	8 (15.4)		Reference
CC+CA	**70 (88.6)**	9 (11.4)	**0.04**^b^	**3.55 (1.03–12.20)**
AA	**12 (66.7)**	6 (33.3)		Reference
				
*VEGF c.-1154G>A*				
GG	43 (84.3)	8 (15.7)	0.71	1.23 (0.39–3.85)
GA+AA	39 (84.8)	7 (15.2)		Reference
GG+GA	78 (85.7)	13 (14.3)	0.28	0.36 (0.05–2.30)
AA	4 (66.7)	2 (33.3)		Reference
				
*VEGF c.-634G>C*				
GG	42 (82.4)	9 (17.6)	0.74	1.21 (0.38–3.83)
GC+CC	40 (87.0)	6 (13.0)		Reference
GG+GC	79 (85.9)	13 (14.1)	0.15	0.23 (0.03–1.70)
CC	3 (60.0)	2 (40.0)		Reference
				
VEGF c.*237C>T				
CC	61 (85.9)	10 (14.1)	0.70	0.79 (0.23–2.66)
CT+TT	21 (80.8)	5 (19.2)		Reference
CC+CT	80 (84.2)	15 (15.8)	0.99	NE
TT	2 (100.0)	0 (0.0)		Reference
				
*VEGFR2 c.-906T>C*				
TT	23 (92.0)	2 (8.0)	0.17	0.33 (0.06–1.63)
TC+CC	59 (81.9)	13 (18.1)		Reference
TT+TC	62 (88.6)	8 (11.4)	0.09	0.37 (0.11–1.92)
CC	20 (74.1)	7 (25.9)		Reference
				
*VEGFR2 c.889G>A*				
GG	58 (84.1)	11 (15.9)	0.87	1.10 (0.31–3.92)
GA+AA	24 (85.7)	4 (14.3)		Reference
GG+GA	81 (85.3)	14 (14.7)	0.36	0.26 (0.01–4.64)
AA	1 (50.0)	1 (50.0)		Reference
				
*GSTM1*				
Present	45 (81.8)	10 (18.2)	0.36	1.73 (0.53–5.67)
Null	37 (88.1)	5 (11.9)		Reference
				
*GSTT1*				
Present	63 (82.9)	13 (17.1)	0.52	1.64 (0.33–8.23)
Null	19 (90.5)	2 (9.5)		Reference
				
*c.-2595C>A+c.-906T>C*				
CC+CA+TT+TC	**53 (88.3)**	7 (11.7)	**0.007**^c^	**9.91 (1.85**–**52.85)**
AA+CC	**3 (37.5)**	5 (62.5)		Reference
				
*c.-1154G>A+c.889G>A*				
GG+GG	31 (86.1)	5 (13.9)	0.59	1.85 (0.19–17.90)
GA+AA+GA+AA	12 (92.3)	1 (7.7)		Reference
				
*c.-634G>C+c.889G>A*				
GG+GG	32 (84.2)	6 (15.8)	0.46	2.29 (0.24–21.51)
GC+CC+GA+AA	14 (93.3)	1 (6.7)		Reference
				
*c.-906T>C+c.889G>A*				
TT+GG	19 (90.5)	2 (9.5)	0.31	0.37 (0.05–2.51)
TC+CC+GA+AA	20 (83.3)	4 (16.7)		Reference
				
				
*c.889G>A+GSTM1*				
GG+Present	32 (82.1)	7 (17.9)	0.37	2.78 (0.29–26.31)
GA+AA+Null	11 (91.7)	1 (8.3)		Reference
				
*VEGF*				
CGGC^d^	**65 (90.3)**	7 (9.7)	**0.02**^e^	**3.86 (1.19**–**12.49)**
Other haplotypes	**17 (68.0)**	8 (32.0)		Reference
				
				
*VEGFR2*				
TG^f^	62 (88.6)	8 (11.4)	0.09	0.37 (0.11–1.19)
Other haplotypes	20 (74.1)	7 (25.9)		Reference

Abbreviations: ASCT, autologous stem cell transplantation; CI, confidence interval; CR, complete response; ISS, International Staging System; *N*, number of patients; NE, not evaluated; OR, odds ratio adjusted by ASCT; PD, progressive disease; PR, partial response; SD, stable disease; VGPR, very good partial response. Significant differences between groups are presented in bold letters.

a The number of patients differed from the total quoted in the study, because it was not possible to obtain pertinent information in some cases.

b *P*_bootstrap_=0.02.

c *P*_bootstrap_=0.002.

d Haplotype of VEGF c.-2595C>A, c.-1154G>A, c.-634G>C and c.*237C>T polymorphisms.

e *P*_bootstrap_=0.01.

f Haplotype of VEGFR2 c.-906T>C and c.889G>A polymorphisms.

**Table 2:** *VEGF*, *VEGFR2*, *GSTM1* and *GSTT1* polymorphisms in survival of multiple myeloma patients

*Variable*	*EFS (*N*=102)*					*OS (*N*=102)*				
	N *of events/*N *total*	*Univariate Cox analysis*		*Multivariate Cox analysis*		N *of events/*N *total*	*Univariate Cox analysis*		*Multivariate Cox analysis*	
		P-*value*	*HR (95% CI)*	P-*value*	*HR (95% CI)*		P-*value*	*HR (95% CI)*	P*-value*	*HR (95% CI)*
*ISS*^a^										
I+II	29/46	0.08	Reference	**0.03**^b^	Reference	19/46	0.08	Reference	0.10^c^	Reference
III	42/55		1.52 (0.94–2.45)		**1.66 (1.03**–**2.70)**	33/55		1.57 (0.89–2.77)		1.59 (0.90–2.80)
										
*ASCT*										
Yes	**20/43**	**< 0.0001**	Reference	**< 0.0001**^d^	Reference	**13/43**	**< 0.0001**	Reference	**< 0.0001**^e^	Reference
No	**51/59**		**3.27 (1.94**–**5.51)**		**3.34 (1.98**–**5.64)**	**39/59**		**3.33 (1.77**–**6.27)**		**3.29 (1.75**–**6.19)**
										
*VEGF c.*-*2595C>A*										
CC	32/49	0.35	0.80 (0.49–1.28)	0.88	0.96 (0.59–1.56)	23/49	0.43	0.80 (0.46–1.39)	0.85	0.95 (0.54–1.66)
CA+AA	39/53		Reference		Reference	29/53		Reference		Reference
CC+CA	59/84	0.95	1.01 (0.54–1.90)	0.74	1.11 (0.58–2.09)	40/84	0.24	0.67 (0.35–1.30)	0.34	0.72 (0.37–1.40)
AA	12/18		Reference		Reference	12/18		Reference		Reference
										
*VEGF c.*-*1154G>A*										
GG	39/55	0.68	1.10 (0.68–1.76)	0.52	1.17 (0.71–1.91)	27/55	0.92	0.97 (0.56–1.68)	0.86	0.95 (0.54–1.67)
GA+AA	32/47		Reference		Reference	25/47		Reference		Reference
GG+GA	68/96	0.58	1.38 (0.43–4.39)	0.84	1.12 (0.35–3.59)	49/96	0.79	0.85 (0.26–2.75)	0.53	0.69 (0.21–2.25)
AA	3/6		Reference		Reference	3/6		Reference		Reference
										
*VEGF c.*-*634G>C*										
GG	37/54	0.77	1.07 (0.67–1.71)	0.78	1.07 (0.66–1.72)	30/54	0.20	1.42 (0.82–2.49)	0.38	1.28 (0.73–2.26)
GC+CC	34/48		Reference		Reference	22/48		Reference		Reference
GG+GC	68/97	0.60	1.36 (0.42–4.39)	0.38	1.69 (0.52–5.50)	49/97	0.97	0.98 (0.30–3.15)	0.74	1.21 (0.37–3.95)
CC	3/5		Reference		Reference	3/5		Reference		Reference
										
VEGF c.*237C>T										
CC	54/76	0.60	1.15 (0.66–1.99)	0.36	1.29 (0.74–2.24)	40/76	0.62	1.17 (0.61–2.25)	0.31	1.39 (0.72–2.68)
CT+TT	17/26		Reference		Reference	12/26		Reference		Reference
CC+CT	70/100	0.61	1.65 (0.22–11.98)	0.40	2.33 (0.31–17.21)	50/100	0.28	0.46 (0.11–1.90)	0.45	0.57 (0.13–2.48)
TT	1/2		Reference		Reference	2/2		Reference		Reference
										
*VEGFR2 c.-906T>C*										
TT	22/28	0.12	1.52 (0.91–2.53)	0.20	1.40 (0.83–2.35)	15/28	0.45	1.25 (0.68–2.30)	0.69	1.12 (0.61–2.07)
TC+CC	49/74		Reference		Reference	37/74		Reference		Reference
TT+TC	54/75	0.14	1.50 (0.86–2.60)	0.05	1.79 (1.02–3.15)	39/75	0.70	1.12 (0.60–2.11)	0.64	1.15 (0.61–2.17)
CC	17/27		Reference		Reference	13/27		Reference		Reference
										
*VEGFR2 c.889G>A*										
GG	**55/73**	**0.006**	**2.22 (1.26**–**3.91)**	**0.001**^f^	**2.62 (1.47**–**4.65)**	**41/73**	**0.04**	**2.00 (1.03**–**3.91)**	**0.02**^g^	**2.21 (1.13**–**4.33)**
GA+AA	**16/29**		Reference		Reference	**11/29**		Reference		Reference
GG+GA	70/100	0.63	1.62 (0.22–11.76)	0.30	2.84 (0.38–20.72)	51/100	0.96	0.95 (0.13–6.95)	0.69	1.48 (0.20–10.91)
AA	1/2		Reference		Reference	1/2		Reference		Reference
										
*GSTM1*										
Present	39/56	0.82	1.05 (0.65–1.68)	0.47	1.18 (0.74–1.89)	33/56	0.10	1.60 (0.91–2.82)	**0.03**^h^	**1.85 (1.04**–**3.28)**
Null	32/46		Reference		Reference	19/46		Reference		Reference
										
*GSTT1*										
Present	55/80	0.46	1.23 (0.70–2.15)	0.97	1.01 (0.57–1.79)	42/80	0.30	1.44 (0.72–2.87)	0.62	1.19 (0.59–2.41)
Null	16/22		Reference		Reference	10/22		Reference		Reference
										
*c.-2595C>A+c.-906T>C*										
CC+CA+TT+TC	47/65	0.65	1.23 (0.49–3.12)	0.22	1.78 (0.70–4.56)	32/65	0.42	0.68 (0.26–1.76)	0.82	0.89 (0.34–2.36)
AA+CC	5/8		Reference		Reference	5/8		Reference		Reference
										
*c.-1154G>A+c.889G>A*										
GG+GG	**30/39**	**0.04**	**2.29 (1.01**–**5.26)**	**0.01**^i^	**2.78 (1.18**–**6.54)**	22/39	0.31	1.59 (0.63–3.95)	0.37	1.52 (0.59–3.90)
GA+AA+GA+AA	**7/13**		Reference		Reference	6/13		Reference		Reference
										
*c.-634G>C+c.889G>A*										
GG+GG	**29/40**	**0.02**	**2.56 (1.14**–**5.73)**	**0.02**^j^	**2.64 (1.15**–**6.05)**	**22/40**	**0.01**	**4.79 (1.42**–**16.15)**	**0.01**^k^	**4.88 (1.42**–**16.70)**
GC+CC+GA+AA	**8/15**		Reference		Reference	**3/15**		Reference		Reference
										
*c.*-*906T>C+c.889G>A*										
TT+GG	**20/24**	**0.002**	**3.34 (1.54**–**7.26)**	**0.002**^l^	**3.48 (1.57**–**7.71)**	13/24	0.06	2.22 (0.94–5.24)	0.08^m^	2.15 (0.90–5.14)
TC+CC+GA+AA	**14/25**		Reference		Reference	9/25		Reference		Reference
										
*c.889G>A+GSTM1*										
GG+Present	**31/40**	**0.04**	**2.21 (1.01**–**4.88)**	**0.01**^n^	**2.80 (1.25**–**6.28)**	**26/40**	**0.02**	**3.30 (1.14**–**9.51)**	**0.008**^o^	**4.23 (1.44**–**12.35)**
GA+AA+Null	**8/13**		Reference		Reference	**4/13**		Reference		Reference
										
*VEGF*										
CGGC^p^	55/77	0.62	1.15 (0.65–2.01)	0.27	1.37 (0.77–2.41)	35/77	0.17	0.66 (0.37–1.19)	0.37	0.76 (0.42–1.38)
Other haplotypes	16/25		Reference		Reference	17/25		Reference		Reference
										
*VEGFR2*										
TG^q^	54/75	0.14	1.50 (0.86–2.60)	0.05	1.79 (1.02–3.15)	39/75	0.70	1.12 (0.60–2.11)	0.64	1.15 (0.61–2.17)
Other haplotypes	17/27		Reference		Reference	13/27		Reference		Reference

Abbreviations: ASCT, autologous stem cell transplantation; CI, confidence interval; EFS, event-free survival; HR, hazard ratio; ISS, International Staging System; *N*, number of patients; OS, overall survival. Significant differences between groups are presented in bold letters.

a The number of patients differed from the total quoted in the study, because it was not possible to obtain pertinent information in some cases.

b *P*_bootstrap_=0.05.

c *P*_bootstrap_=0.10.

d *P*_bootstrap_=0.001.

e *P*_bootstrap_=0.001.

f *P*_bootstrap_=0.005.

g *P*_bootstrap_=0.02.

h *P*_bootstrap_=0.04.

i *P*_bootstrap_=0.01.

j *P*_bootstrap_=0.03.

k *P*_bootstrap_=0.005.

l *P*_bootstrap_=0.003.

m *P*_bootstrap_=0.08.

n *P*_bootstrap_=0.01.

o *P*_bootstrap_=0.01.

p Haplotype of *VEGF* c.-2595C>A, c.-1154G>A, c.-634G>C and c.*237C>T polymorphisms.

q Haplotype of *VEGFR2* c.-906T>C and c.889G>A polymorphisms. In multivariate Cox analysis adjusted by ISS and ASCT.
