# Supplementary material for: VEGF, VEGFR2 and GSTM1 polymorphisms in outcome of multiple myeloma patients treated with thalidomide-based regimens
Source: Blood Cancer J. 2017 Jun 30;7(6):e580–. doi: 10.1038/bcj.2017.58 (PMC5520405; doi:10.1038/bcj.2017.58)
Supplement: Supplementary Table S1 [file bcj201758x1.doc]

**Table S1**. Clinicopathological aspects of multiple myeloma patients.

| **Variable** | **N (%) or median (range)** |
| --- | --- |
| **Age (years)** | 63 (36-82) |
| **Gender** |  |
| Male | 52 (51.0) |
| Female | 50 (49.0) |
| **Ethnic origin** |  |
| Caucasian | 96 (94.1) |
| Non-Caucasian | 6 (5.9) |
| **Bone marrow plasma cells (%)** | 31 (10-93) |
| **M-component**^*^ |  |
| IgG | 55 (59.1) |
| IgA | 16 (17.2) |
| Light chain | 22 (23.7) |
| **Type of light chain** |  |
| Kappa | 11 (50.0) |
| Lambda | 11 (50.0) |
| **Creatinine (mg/dL)** | 1.2 (0.3-13.1) |
| **Hemoglobin (g/dL)** | 9.6 (6.1-16.5) |
| **Calcium (mg/dL)** | 9.3 (6.1-16.5) |
| **Proteinuria (g/24-hour)** | 0.4 (0.01-23.1) |
| **Lactate dehydrogenase (U/L)** | 306.0 (138.0-1060.0) |
| **β2-microglobulin (mg/L)** | 5.9 (1.7-69.0) |
| **Albumin (g/dL)** | 3.5 (1.3-5.1) |
| **ISS**^*^ |  |
| I | 17 (16.8) |
| II | 29 (28.7) |
| III | 55 (54.5) |
| **Treatment scheme** |  |
| Cyclophosphamide-thalidomide-dexamethasone | 45 (44.1) |
| Thalidomide-dexamethasone | 34 (33.4) |
| Melphalan-prednisone-thalidomide | 18 (17.6) |
| Bertozomib-thalidomide-dexamethasone | 5 (4.9) |
| **ASCT** |  |
| Yes | 43 (42.2) |
| No | 59 (57.8) |

(N) number of patients; (^*^) the number of patients differed from the total quoted in the study, because it was not possible to obtain M-component and tumor stage in some patients; (ISS) International Staging System; (ASCT) autologous stem cell transplantation.
