# Supplementary figures and images for: VEGF, VEGFR2 and GSTM1 polymorphisms in outcome of multiple myeloma patients treated with thalidomide-based regimens
Source: Blood Cancer J. 2017 Jun 30;7(6):e580–. doi: 10.1038/bcj.2017.58 (PMC5520405; doi:10.1038/bcj.2017.58)

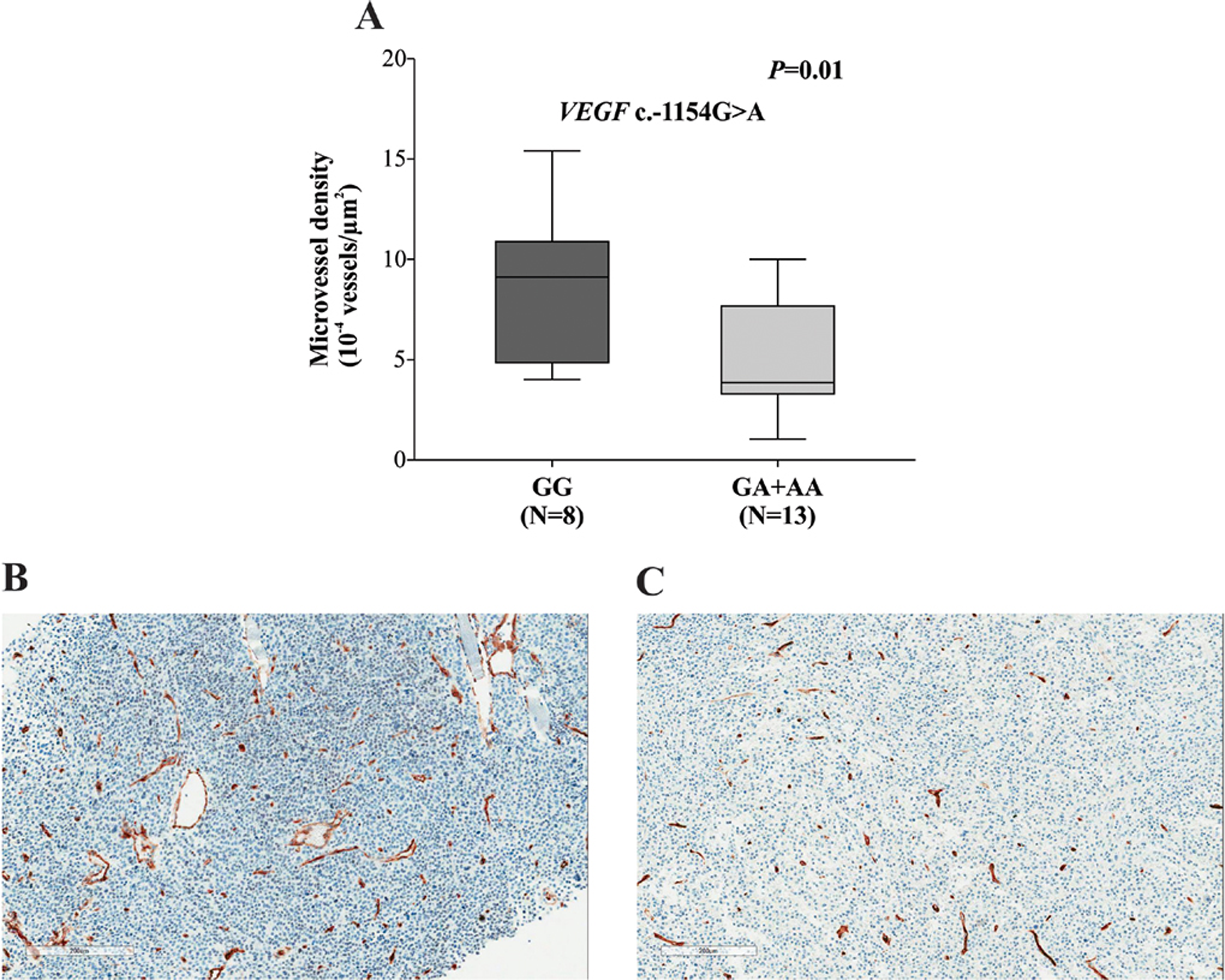

Supplement: Supplementary Figure S1 [file bcj201758x2.tif]

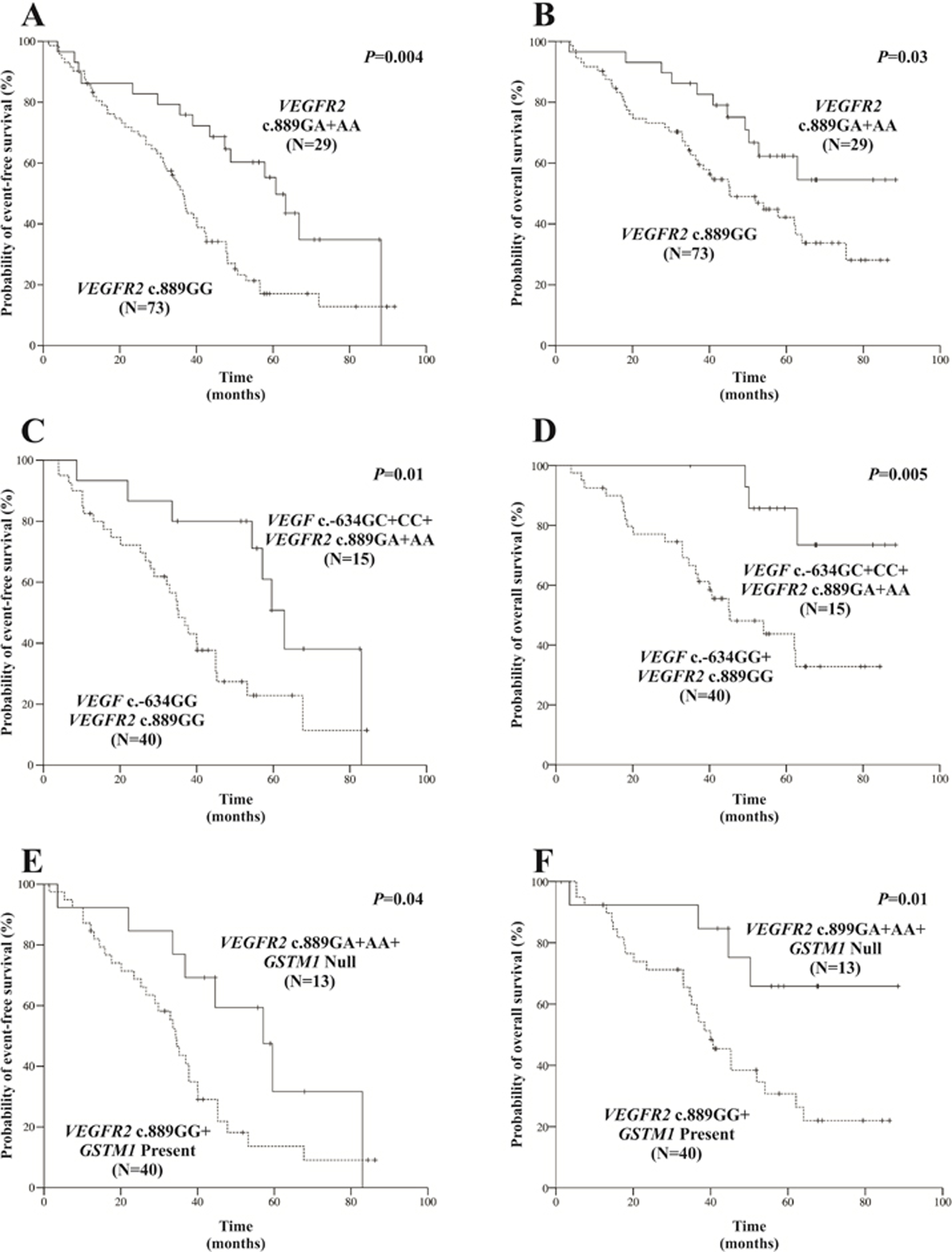

Supplement: Supplementary Figure S2 [file bcj201758x4.tif]
