# Supplementary material for: VEGF, VEGFR2 and GSTM1 polymorphisms in outcome of multiple myeloma patients treated with thalidomide-based regimens
Source: Blood Cancer J. 2017 Jun 30;7(6):e580–. doi: 10.1038/bcj.2017.58 (PMC5520405; doi:10.1038/bcj.2017.58)
Supplement: Supplementary Figure S1 Legend [file bcj201758x3.doc]

**Figure S1**. (**A**) Microvessel density measured by CD34 staining in bone marrow fragments of multiple myeloma patients stratified by *VEGF* c.-1154G>A genotypes (GG *vs*. GA or AA; *P*= 0.01). (**B**, **C**) Extreme images of cases with the *VEGF*
c.-1154GG and AA genotypes with high and low microvascular densities, respectively.
