# Supplementary material for: VEGF, VEGFR2 and GSTM1 polymorphisms in outcome of multiple myeloma patients treated with thalidomide-based regimens
Source: Blood Cancer J. 2017 Jun 30;7(6):e580–. doi: 10.1038/bcj.2017.58 (PMC5520405; doi:10.1038/bcj.2017.58)
Supplement: Supplementary Figure S2 Legend [file bcj201758x5.doc]

**Figure S2**. Probabilities of event-free and overall survival of multiple myeloma patients treated with thalidomide-based regimens and stratified by *VEGFR2* c.889G>A (**A**, **B**), *VEGF* c.-634G>C plus *VEGFR2* c.889G>A (**C**, **D**), and *VEGFR2* c.889G>A plus *GSTM1* (**E**, **F**) genotypes.
